# Modulation of Cox-1, 5-, 12- and 15-Lox by Popular Herbal Remedies Used in Southern Italy Against Psoriasis and Other Skin Diseases

**DOI:** 10.1002/ptr.5234

**Published:** 2014-10-03

**Authors:** Ammar Bader, Francesca Martini, Guillermo R Schinella, Jose L Rios, Jose M Prieto

**Affiliations:** 1 Department of Pharmacognosy, Faculty of Pharmacy, Umm Al-Qura UniversityMakkah, 21955, Saudi Arabia; 2 Departament de Farmacologia, Facultat de Farmacia, Universitat de Valencia46100, Burjassot, Valencia, Spain; 3 Catedra de Farmacologia Basica, Facultad de Ciencias Medicas, Universidad Nacional de La Plata, CIC Provincia de Buenos Aires1900, La Plata, Buenos Aires, Argentina; 4 Centre for Pharmacognosy and Phytotherapy, University College London School of PharmacyWC1N 1AX, London, United Kingdom

**Keywords:** herbal medicine, psoriasis, leukotrienes, prostaglandins, Asteraceae, Acanthaceae

## Abstract

*Acanthus mollis* (Acanthaceae), *Achillea ligustica*, *Artemisia arborescens* and *Inula viscosa* (Asteraceae) are used in Southern Italy against psoriasis and other skin diseases that occur with an imbalanced production of eicosanoids. We here assessed their *in vitro* effects upon 5-, 12-, 15-LOX and COX-1 enzymes as well as NFκB activation in intact cells as their possible therapeutic targets. All methanol crude extracts inhibited both 5-LOX and COX-1 activities under 200 µg/mL, without significant effects on the 12-LOX pathway or any relevant *in vitro* free radical scavenging activity. NFκB activation was prevented by all extracts but *A. mollis*. Interestingly, *A. ligustica*, *A. arborescens* and *A. mollis* increased the biosynthesis of 15(*S*)-HETE, an anti-inflammatory eicosanoid. *A. ligustica* (IC_50_ = 49.5 µg/mL) was superior to *Silybum marianum* (IC_50_ = 147.8 µg/mL), which we used as antipsoriatic herbal medicine of reference. Its *n*-hexane, dichloromethane and ethyl acetate fractions had also inhibitory effects on the LTB_4_ biosynthesis (IC_50_s = 9.6, 20.3 and 68 µg/mL, respectively) evidencing that the apolar extracts of *A. ligustica* are promising active herbal ingredients for future phytotherapeutical products targeting psoriasis. © 2014 The Authors. *Phytotherapy Research* published by John Wiley & Sons Ltd.

## Introduction

Psoriasis is one of the most important chronic pathologies of the skin, affecting roughly 1–3% of the world's population. Apart from being usually recurrent, psoriasis can often be very debilitating, with 5–10% of all patients developing psoriatic arthritis, which causes inflammation and swelling in the hands, feet and large joints (DiSepio *et al.*, 1999). As the skin is the most accessible and largest of our organs, remedies for the many dermatological disorders have been well developed—and are consistently preserved—within traditional health systems.

Ethnobotanical investigations in Sicily reported the use of several herbal remedies for the relief of psoriasis symptoms. These included ‘biancarussina’ (*Acanthus mollis* L., Acanthaceae), ‘millefoglio’ (*Achillea spp*, Asteraceae), ‘erva bianca’ (*Artemisia arborescens* L., Asteraceae) and ‘vrucara’ (*Inula* v*iscosa* (L.) Aiton, Asteraceae). These plants are collected during spring time and used to make a decoction by crushing and soaking their aerial parts in cold water and untreated wine (Amenta *et al.*, 2000). The use of *A. mollis* and *Achillea ligustica* is also reported as a first choice herbal remedy for skin diseases in zones of Sardinia (Bruni *et al.*, 1997) and the decoction of the roots of *I. viscosa* is used in Calabria—under the local name of ‘spulitru’—for the relief of skin irritation of allergic origin (Passalacqua *et al.*, 2007).

Eicosanoids have been assigned an important role in inflammatory skin diseases such as psoriasis and atopic dermatitis. Neutrophils rapidly migrate into the inflamed epidermis and get into close contact with the keratinocytes releasing leukotriene A_4_ (LTA_4_) into the extracellular space. From this substrate, transcellular leukotriene synthesis may therefore be an important mechanism by which the human epidermis can contribute significantly to leukotriene B_4_ (LTB_4_) formation. In addition, 12(*S*)-hydroxy-5*Z*,8*Z*,10*E*,14*Z*-tetraenoic acid (12(*S*)-HETE) has been shown as one of the main eicosanoids formed by the epidermis and has been detected in large quantities in human psoriatic scales (Iversen and Kragballe, 2000). Thus, the putative activity of extracts prescribed for such disorders may arise—at least in part—from their ability to inhibit—directly or indirectly—the activity of the enzymes responsible for their synthesis.

Some aspects of the phytochemistry and pharmacology of these species have been already published elsewhere, but to the best of our knowledge, there is not a comparative assessment of their actions on COX and LOX pathways in intact pro-inflammatory mammalian cells yet. We therefore aim to assess the *in vitro* effect of these herbal remedies on the release of LTB_4_, other major pro- and anti-inflammatory eicosanoids and the activation of the nuclear factor kappaB (NFκB). Their antioxidant activity was tested just to rule out possible unspecific inhibition of COX and LOX enzymes, which are redox-sensitive enzymes. We hope in this manner to gain insight into the possible biochemical mechanisms of these extracts that justify their popular antipsoriatic use in Southern Italy. This could facilitate the development of rational formulations in herbal medicinal products against inflammatory topical conditions.

## Material and Methods

**Chemicals.** *Boswellia serrata* H15® resin was obtained from Gufic Chemicals (India). It consists on a standardised extract rich in the 5-LOX inhibitor acetyl-11-keto-β-boswellic acid (Glaser *et al.*, 1999). All other chemicals were of the highest available analytical grade and purchased from Sigma-Aldrich (USA) or from Merck (Germany). Solvents (HPLC grade) were provided by JT Baker (The Netherlands) and Fisher (UK).

**Biological material.** Buffy coats were obtained from the Centre de Transfusions de la Generalitat Valenciana (Valencia, Spain). Wistar rats, each 250–300 g, were provided by the animal facility of the Faculty of Pharmacy (University of València). Housing conditions and all *in vivo* experiments were approved by the institutional Ethical Committee of the Faculty of Pharmacy, University of Valencia (Spain), according to the guidelines established by the European Union on Animal Care (CEE Council 86/609).

**Plant material.** The leaves of *A. mollis* L. (Acanthaceae), *A. ligustica* L. (Asteraceae), *A. arborescens* L. (Asteraceae) and the flowering aerial parts of *Inula viscosa* (L.) Aiton (Asteraceae) were collected and identified by one of the co-authors (A.B.) in Novara di Sicilia (Messina, Italy, Spring 1998). Voucher specimens (references 3563–Pharm, 3982-Pharm 4027–Pharm and 3758-Pharm, respectively) were deposited at the herbarium of the Dipartimento di Chimica Bioorganica e Biofarmacia, Universita degli Studi di Pisa (Italy). Milk thistle (*Silybum marianum* L.) of pharmacopoeial grade was supplied by Professor J. B. Peris (Department of Botany, Faculty of Pharmacy, University of Valencia, Spain).

**Preparation of extracts.** The powdered dried drugs were macerated in methanol (20 g in 400 mL, 24 h). The solvent was evaporated under vacuum, and the residues were lyophilised. The dried methanolic extract of *A. ligustica* (2.70 g) was dissolved in 10% aqueous methanol and partitioned with solvents of increasing polarity (*n*-hexane, dichloromethane, ethyl acetate and *n*-butanol) yielding four fractions (M = 622, 695, 165 and 380 mg, respectively).

For the *in vitro* assays, the plant extracts were dissolved in dimethyl sulfoxide (DMSO) by vigorous shaking for 30 min. Non soluble parts were removed by centrifugation (10 min, 10000 ×*g*), and the stock solutions were adjusted to 40 mg/mL. The final concentration of DMSO was 0.05% in all incubations including controls.

**Solid phase extraction (SPE) and analytical HPLC system.** Solid phase extraction (SPE) was performed with Lichrolut® columns C18, 100 mg, 1 mL (Merck) attached to a 12-port vacuum manifold Visiprep® (Supelco). HPLC-DAD analysis of eicosanoids was performed on a Merck-Hitachi system consisting on an Intelligent Pump L-6200, Diode Array Detector L-7455, Auto Sampler L-7200, precolumn Lichrospher® C18 (4 × 4 mm, 5 µm, Merck), column Lichrospher® C18 (250 × 4 mm, 5 µm Merck) and software HSM-7000. Elution conditions were as follows: flow rate 1 mL/min; 0–27 min, 100% A; 27.1–27.6 min, 0% A; 27.7–40 min, 100% A; Eluents MeOH/H_2_O (74:26) + trifluoroacetic acid (0.007%) (A) and MeOH (B). All plant extracts were fingerprinted using a Waters system consisting on a series 600 pump, Series 990 Diode array, column Phenomenex® C18 (250 × 4 mm, 5 µm), and processed using the Millenium software. Chromatograms and elution conditions for each one are available as supplementary materials.

**Cytotoxicity assay.** The 3-(4,5-dimethylthiazol-2-yl)-2,5-diphenyl tetrazolium bromide (MTT) assay described by Mosmann (1983) was used as a criterion of cytotoxicity. Human leukocytes (1 × 10^6^ cells) were preincubated at 37°C for 30 min in Dulbecco's phosphate buffered saline (PBS) pH 7.4, containing the extracts at 200 µg/mL. The dark blue formazan coloured metabolite was dissolved in DMSO in an ultrasonic bath and measured at 490 nm.

**Assay of 5-LO activity.** Rat polymorphonuclear leukocytes (PMNL) were harvested by intraperitoneal injection of glycogen. The elicited PMNL (5 × 10^6^) were incubated and processed as described by Safayhi *et al.* (1995). Leukotriene B_4_ (LTB_4_) was selectively extracted from the cell supernatants by SPE and quantified by HPLC-DAD with the help of the internal standard Prostaglandin B_2_ (PGB_2_).

**Assay of COX-1, 12-, and 15-LOX activities.** Human platelets were obtained from human buffy-coats. A differential counting was done using a Coulter Counter (Sysmex D-800, Kobe, Japan). Their viability was assessed by fluorescence microscopy (Nikon, Japan) staining with acridine orange/ethidium bromide. Aliquots of 80 × 10^6^ platelets were incubated and processed as described by Safayhi *et al.* (1992). Absolute quantification of 12(*S*)-hydroxy-5Z,8E,10E-heptadecatrienoic acid (12(*S*)-HHTrE) from the COX-1 pathway, 12(*S*)-hydroxy-5Z,8Z,10E,14Z-eicosatetraenoic acid (12(*S*)-HETE) from the 12-LOX pathway and 15(*S*)-hydroxy-5Z,8Z,11Z,13E-eicosatetraenoic acid (5(*S*)-HETE) acid was performed by means of external and internal standards. Phospholipase A_2_ (PLA_2_) impairment can be also indirectly estimated in this model as discussed in the next section.

**Assay of the activation of the NFκB.** HeLa-luc cells, consisting on HeLa cells expressing a luciferase reporter gene controlled by the IL-6 promoter, were kindly provided by Prof. M. Heinrich (UCL School of Pharmacy). IL-6 is one of the target genes for activated NFκB. Therefore, the chemiluminescence produced by luciferase can be measured as an IL-6 dependent measurement of the activation or inhibition of NFκB, as described by Bremner *et al.* (2004).

**Redox properties assays.**For the ABTS^•+^ radical decolorization, 10 μL of a solution of the extracts (10–100 µg/mL) in water was added to 1 mL of ABTS^•+^ solution, and the absorbance at 734 nm was determined after 30 min (Pannala *et al.*, 2001). For the DPPH scavenging assay, 100 μL of a DPPH solution (0.1 μM) was added to 100 μL of a methanolic solution of the extracts. After 30 min the absorbance at 540 nm was measured (Brand-Williams *et al.*, 1995). All incubations were conducted in the dark at room temperature.

**Statistical methods.** Percentages of inhibition are shown as mean ± S.E.M. of three or more independent experiments, and every experiment was performed in duplicate. The inhibition of 5-LOX total activity is expressed as percentages of LTB_4_ released with respect to the control. Inhibition of COX-1 and 12-LOX activities is expressed as percentages with respect to the control of 12(*S*)-HHTrE and 12(*S*)-HETE), respectively. Linear regressions and statistical evaluation were performed by ANOVA followed by Dunnett's *t*-test for multiple comparisons using Graph-Pad InStat 3.0 and Prism 4.0 software. Values with *p* <; 0.05 were considered significant.

## Results

### Extraction of plant material

Yields of the extractions were: *A. mollis*, leaves, 24.4%; *A. ligustica*, leaves, 13.5%; *A. arborescens*, leaves, 13.1%; *I. viscosa*, aerial parts, 9.3%.

### Cytotoxicity

None of the extract resulted cytotoxic to rat PMNs or human platelets at the assayed doses as per the MTT assay (data not shown).

### Effects on 5-LOX activity

When tested at 200 µg/mL, the methanolic extracts of *A. ligustica*, *A. arborescens* and *I. viscosa* completely inhibited the LTB_4_ biosynthesis (100%) as evidenced by the absence of any significant peak corresponding to LTB_4_. *S. marianum* extract achieved a 61% of reduction only (*p* <; 0.001). *A. mollis* methanol extract resulted the less active (30%) but was still highly significant (*p* <; 0.001).

**Figure 1:**
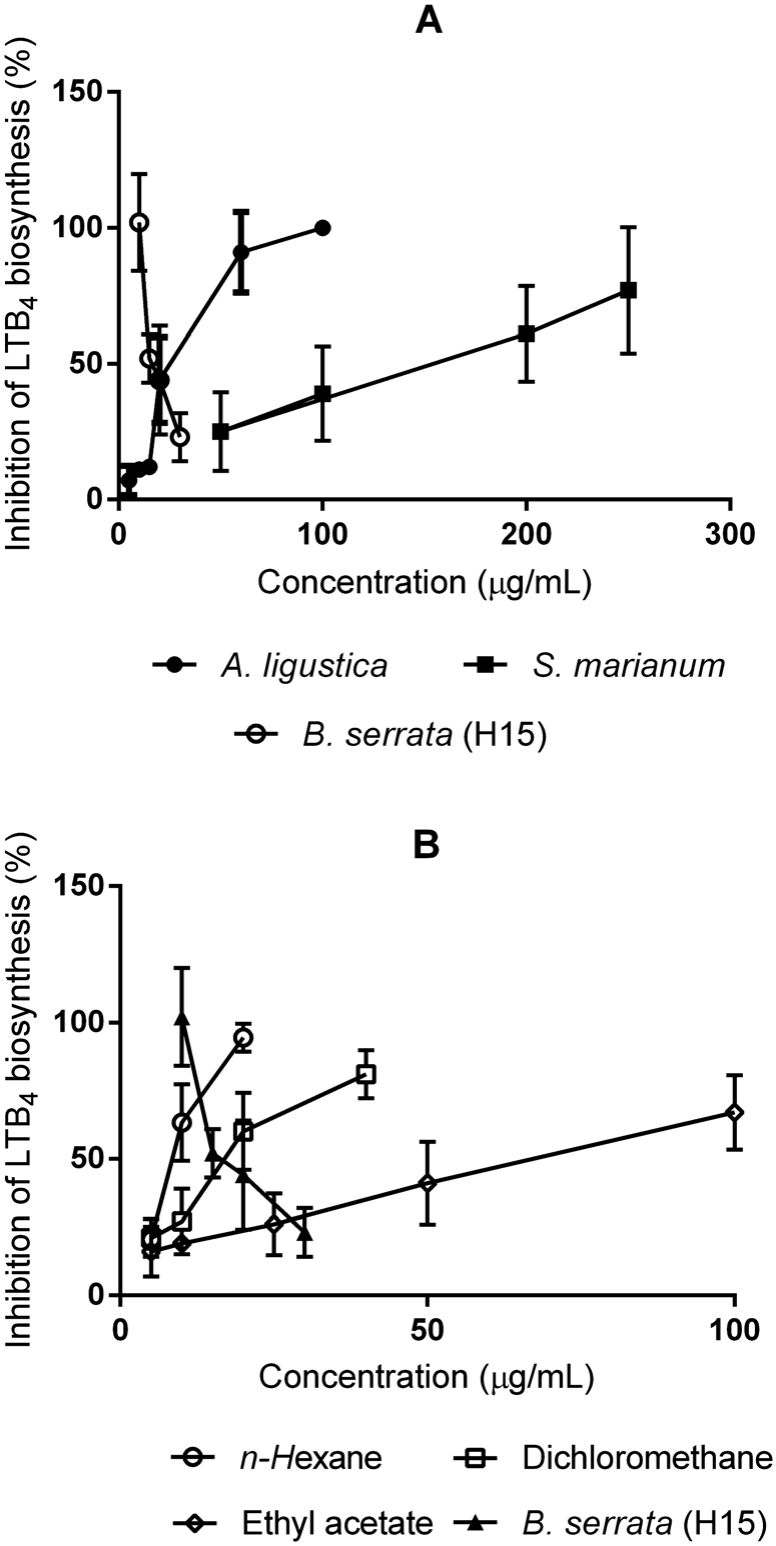
Effect of crude methanolic plant extract (A) and fractions (B) of *Achillea ligustica* vs. *Silibum marianum* and *Boswellia serrata* H15© extracts on the biosynthesis of LTB4 in intact peritoneal PMNs (mean ± SD).

Unfortunately, the calculation of the IC_50_ was only possible for the methanolic extract of *A. ligustica* (IC_50_ = 49.5 µg/mL) (Fig. 1A) because the secondary metabolites of *A. arborecens* and *I. viscosa* co-eluted with the internal standard (PGB_2_). However, their IC_50_ are lower than 100 µg/mL, thus resulting more active than *S. marianum* (IC_50_ = 147.8 µg/mL). The IC_50_ for *A. ligustica* is comparable to the commercial product H15®, an extract enriched in boswellic acids that are specific, non redox inhibitors of LTB_4_ biosynthesis (Glaser *et al.*, 1999; Safayhi *et al.*, 1992).

When *A. ligustica* was further subjected to a liquid–liquid fractionation, the resulting *n*-hexane, dichloromethane and ethyl acetate fractions also had inhibitory effect on the LTB_4_ biosynthesis with IC_50_ of 9.6 µg/mL, 20.3 µg/mL and 68 µg/mL, respectively (Fig. 1B), while the *n*-butanol fraction was inactive at the maximum tested concentration of 200 µg/mL.

### Effects on COX-1, 12- and 15-LOX activities

**Figure 2:**
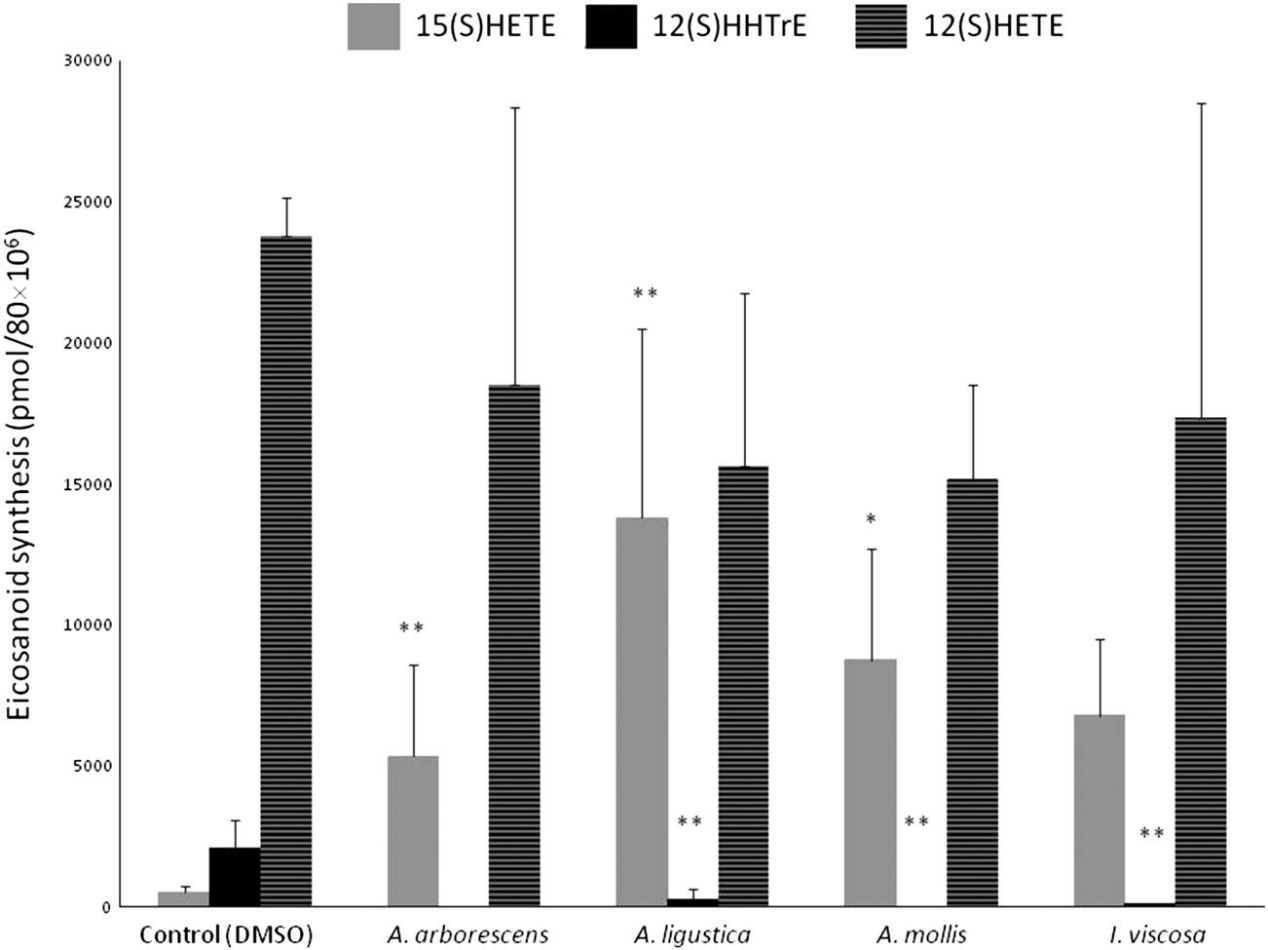
Effects of the crude methanolic extracts (200 µg/mL) on the biosynthesis of 15(S)-HETE (grey left bar), 12(S)-HHTrE (black middle bar) and 12(S)-HETE (stripped right bar) by human platelets (mean ± SD). ANOVA followed by Dunnett's test; (*) *p* <; 0.05; (**) *p* <; 0.001.

The crude plant extracts were tested at 200 µg/mL in human platelets (*n* = 3) (Fig. 2). They all resulted potent inhibitors of the COX-1 activity measured as 12(*S*)-HHTrE. We also noticed that extracts of *A. ligustica* and *A. mollis* significantly enhanced the biosynthesis of 15(*S*)-HETE in human platelets. However, none was able to inhibit 12-LOX activity. We used piroxicam (50 ± 4% inhibition at 6.25 μM) for COX-1 activity and *nor*-dihydroguaiaretic acid (NDGA) for COX-1 and 12-LOX activity (46 ± 3% and 59 ± 8% inhibition at 10 μM, respectively) as reference drugs. Because our experimental model does not add exogenous arachidonic acid (AA), an impairment of the PLA_2_ activity can be ruled out.

### Antioxidant activity

**Figure 3:**
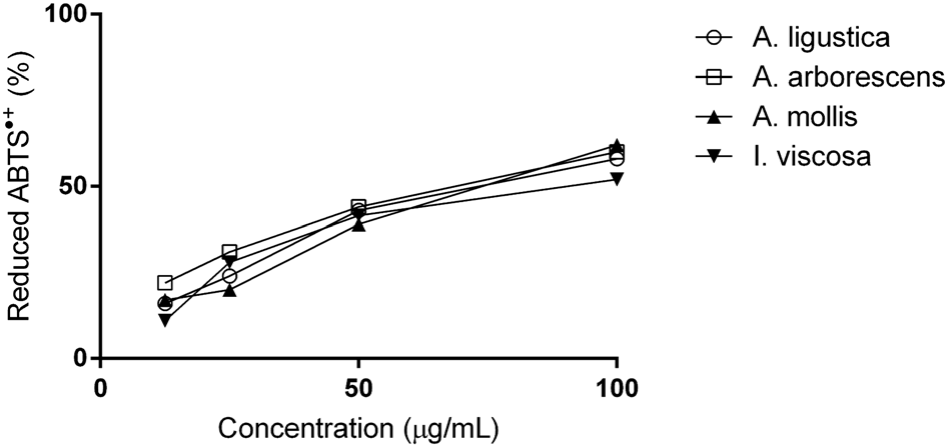
Free radical scavenging activity of the plant extracts in the ABTS assay (mean ± SD).

The IC_50_ values of the free radical scavenger activity of the extracts ranged 71–83 µg/mL in the ABTS^•+^ method (Fig. 3) whilst they were higher than 100 µg/mL in the DPPH method (data not shown). We consider these values quite modest, and we do not think that antioxidant activities contribute to the *in vitro* anti-inflammatory properties of these extracts. Gallic acid was used as reference compound (IC_50 ABTS_ = 1.9 μM; IC_50 DPPH_ = 4.7 μM).

### Activation of the NFκB

**Figure 4:**
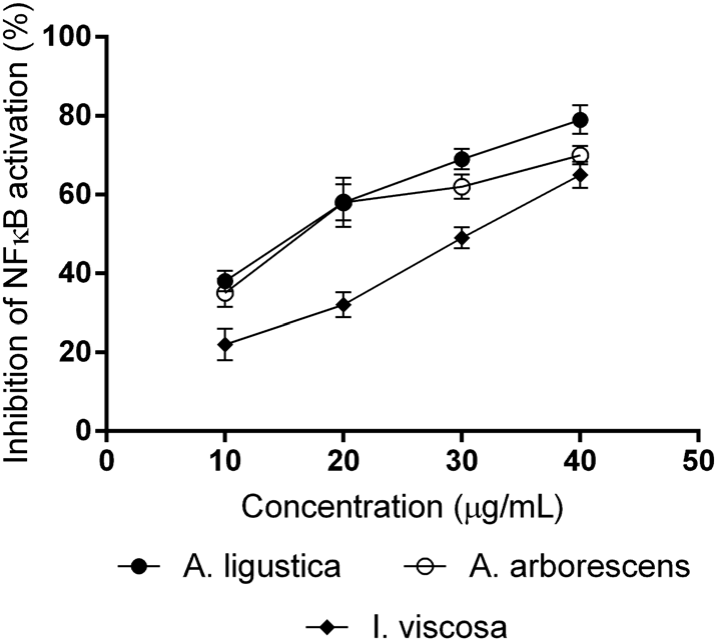
Inhibitory effect of the plant extracts on NFκB activation (mean ± SD).

*A. ligustica* (IC_50_ = 16.7 µg/mL), *A. arborescens* (IC_50_ = 19.2 µg/mL) and *I. viscosa* (IC_50_ = 30.4 µg/mL) resulted very active preventing the activation of this important nuclear factor (Fig. 4). On the other hand, *A. mollis* resulted inactive (IC_50_ ≥ 200 µg/mL). Parthenolide (57 ± 4% inhibition at 5 μM) was used as reference drug.

## Discussion

The selected popular Italian antipsoriatic herbal medicines are endowed with dual COX-1 and 5-LOX inhibitory activity. The search for dual inhibitory activity in anti-inflammatory medicines is regarded as the next step in the evolution of therapeutic agents in psoriasis, among other chronic conditions (Charlier and Michaux, 2003). These plant extracts did not show any *in vitro* relevant activity as radical scavengers, which may rule out unspecific antioxidant inhibition of the redox sensitive COX and LOX enzymes.

On the other hand, the activity of the proinflammatory enzyme 12-LOX was not inhibited by any of the plant extracts below 200 µg/mL. Only *A. mollis* had a borderline inhibitory effect on this enzyme, and slightly higher concentrations may well turn off the biosynthesis of 12(*S*)-HETE. As previously mentioned, 12(*S*)-HETE is present in psoriatic scales (Iversen and Kragballe, 2000). Human platelets produce 12(*S*)-HHTrE and 12(*S*)-HETE from the COX-1 and 12-LOX pathways, respectively, after stimulation with Ca^++^ and ionophore A23187. In psoriatic epidermis only the platelet-type 12-LOX is detectable (Henke *et al.*, 1986) so this *in vitro* model is relevant for the screening for antipsoriatic drugs.

*A. ligustica* and *A. mollis* extracts enhance the biosynthesis of 15(*S*)-HETE, an anti-inflammatory eicosanoid (Fig. 2). The synthesis of anti-inflammatory autacoids such as lipoxins and resolvins also depends on the 15-LOX enzyme (Serhan *et al.*, 2008; Sun *et al.*, 2007). The 15-LOX enzyme is a minor pathway in human platelets stimulated with ionophore A23187, and the exogenous or endogenous AA is channelled primarily to the formation of other eicosanoids (Spector *et al.*, 1988). In this context, the overproduction of 15(*S*)-HETE is likely due to a phenomenon known as *shunting*, consisting on the channelling of the endogenous AA that is not metabolized by COX-1 and 12-LOX into the 15-LOX pathway. One of the pharmacological effects of such an increase could actually be the enhancement of the anti-inflammatory activity, since 15(*S*)-HETE is itself an inhibitor of both 12-LOX and PLA_2_ activities in human platelets (Spector *et al.*, 1988).

In addition, *A. ligustica*, *A. arborescens* and *I. viscosa* are endowed with NFκB inhibitory activity. This activity has been linked—at least in part—to the presence of sesquiterpene lactones. This nuclear factor is able to inhibit the expression of genes encoding the enzymes responsible for the eicosanoids biosynthesis (Yamamoto and Gaynor, 2004). However, it was demonstrated that intact cell models of eicosanoids biosynthesis using short incubations—similar to those performed in this work—reflect the direct interaction of the secondary metabolites with the arachidonate cascade rather than any effect on the NFκB (Tornhamre *et al.*, 2001). Therefore our overall results are showing a potent, specific and immediate effect of the plant extracts upon the enzymes involved in the arachidonate pathway, which is followed at a later stage by a NFκB inhibitory activity.

We also conducted a comparison of *A. ligustica* with two antipsoriatic herbal medicines of reference. The use of *S. marianum* L. across Europe as an antipsoriatic remedy is well documented (Amenta *et al.*, 2000). However, Milk Thistle has been better known as a secular remedy for hepatic disorders in Western phytotherapy, which is underpinned by its antioxidant, radical scavenger, anti-inflammatory and immunostimulant properties (Flora *et al.*, 1998; Luper, 1999). The use of this species as antipsoriatic is believed to be due to its content on silibinin (Dehmlow *et al.*, 1996), a potent inhibitor of LTB_4_ biosynthesis by leukocytes. In fact, leukotrienes are an important therapeutic target in psoriasis (Voorhees, 1983). In this regard the resin of *B. serrata* L. and its active principle acetyl-11-keto-beta boswellic acid (AKBA), a known potent, direct, non-redox 5-LOX inhibitor (Safayhi *et al.*, 1995), is another herbal remedy of Ayurveda origin with an enormous potential in the therapy of psoriasis (Ammon *et al.*, 1993; Wang *et al.*, 2009). In terms of the inhibition of leukotriene biosynthesis, *A. ligustica* crude extract resulted more active than *S. marianum*, and almost equal to H15®, a proprietary formulation of *B. serrata* enriched in AKBA (Glaser *et al.*, 1999) (Fig. 1A).

The apolar fractions of *A. ligustica* retain—and enhance—the 5-LOX inhibitory activity. This was evident after L/L fractionation of the crude methanol extract of *A. ligustica*, with the hexanic fraction resulting highly active, and the dichlorometane fraction being of similar activity to H15® (Fig. 1B). Previous works showed that these apolar extracts may contain alkamides, which are reported to be good inhibitors of both COX and 5-LOX *in vitro* (Muller-Javic et al., 1994) and volatiles (Bader *et al.*, 2007). Still we should not dismiss a contribution of more polar sesquiterpene lactones and flavonoids present in the crude extract which are also characteristic of *Achillea* species (Bruno, 1988; Tzakou *et al.*, 1995).

## Conclusion

We here demonstrate that the simultaneous, acute, direct inhibition of 5-LOX and COX-1 enzymatic pathways, complemented with an anti-inflammatory activity at the level of the NFκB in the case of the Asteraceae, is an important mechanism underlying the popular use of reported antipsoriatic plant species in Southern Italy. *A. ligustica*, *A. arborescens* and *A. mollis* are of particular interest as they also enhance the biosynthesis of 15(*S*)-HETE—an antiinflammatory eicosanoid. The crude and apolar extracts of *A. ligustica* were more active as 5-LOX inhibitors than pharmacopoeial and proprietary extracts of reference in the phytotherapy of psoriasis, making them potential active ingredients for future formulations of novel anti-psoriatic herbal medicinal products.
