# Supplementary figures and images for: Modulation of Cox-1, 5-, 12- and 15-Lox by Popular Herbal Remedies Used in Southern Italy Against Psoriasis and Other Skin Diseases
Source: Phytother Res. 2014 Oct 3;29(1):108–13. doi: 10.1002/ptr.5234 (PMC4303945; doi:10.1002/ptr.5234)

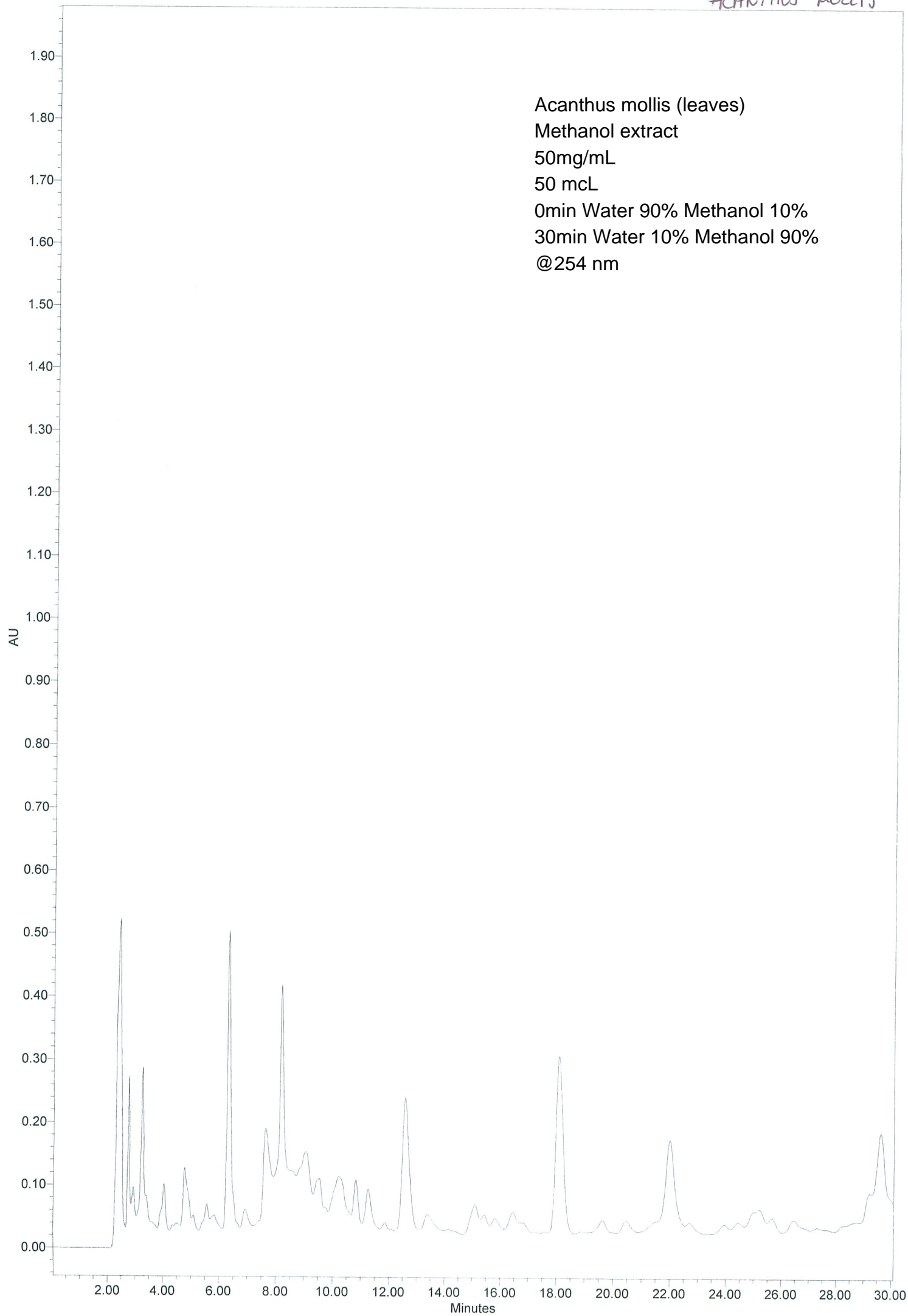

*Achillea ligustica*

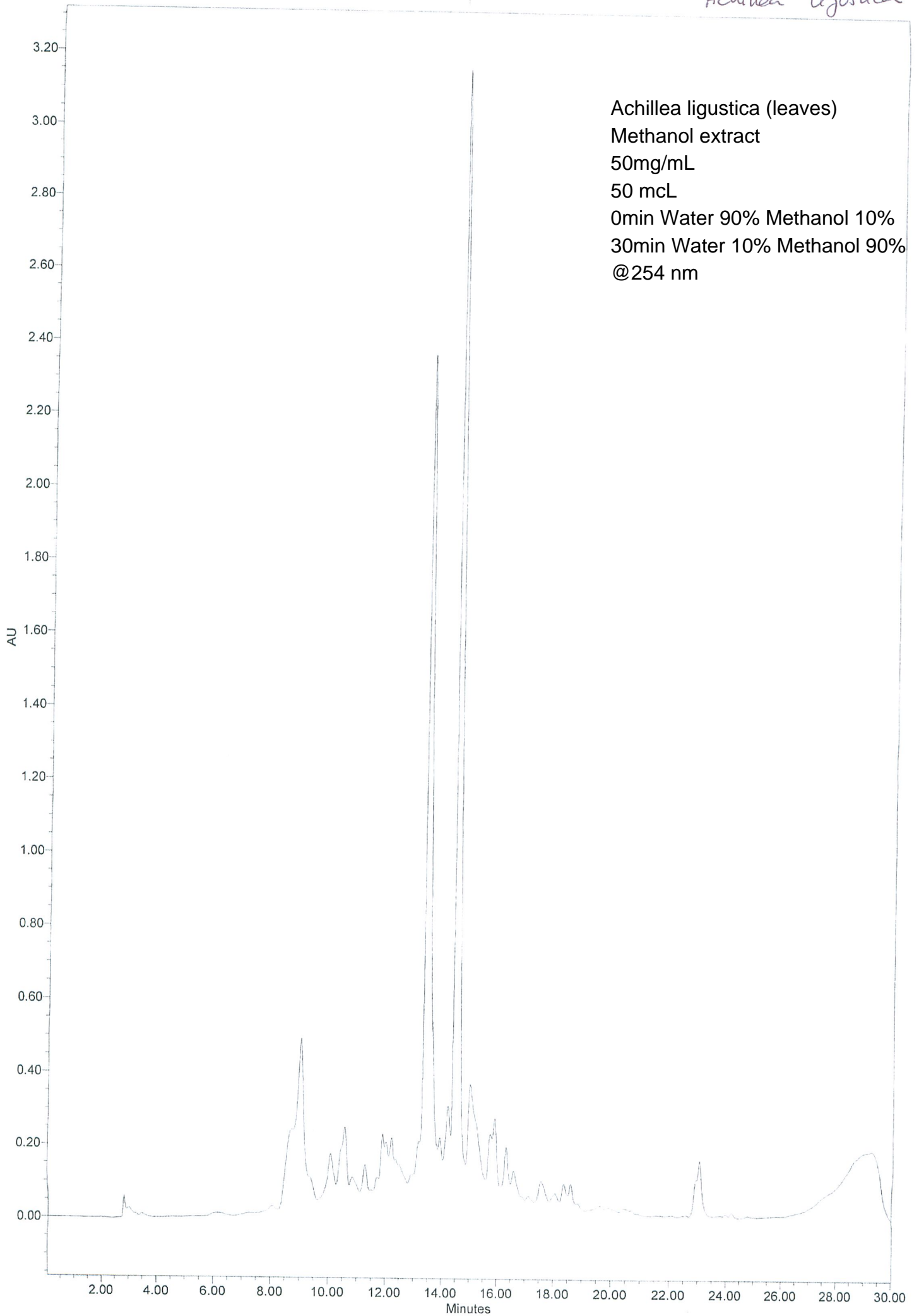

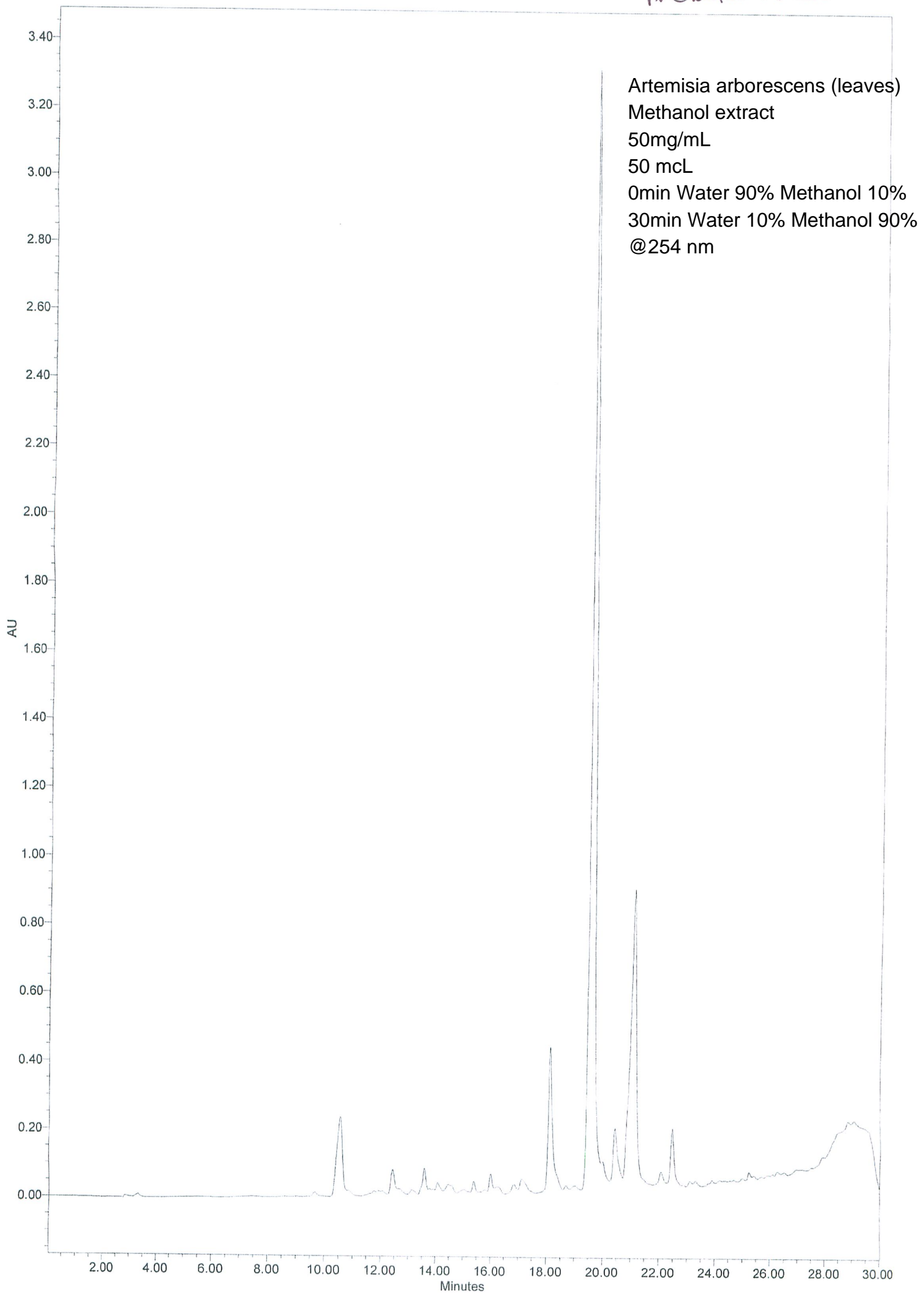

*Inula viscosa*

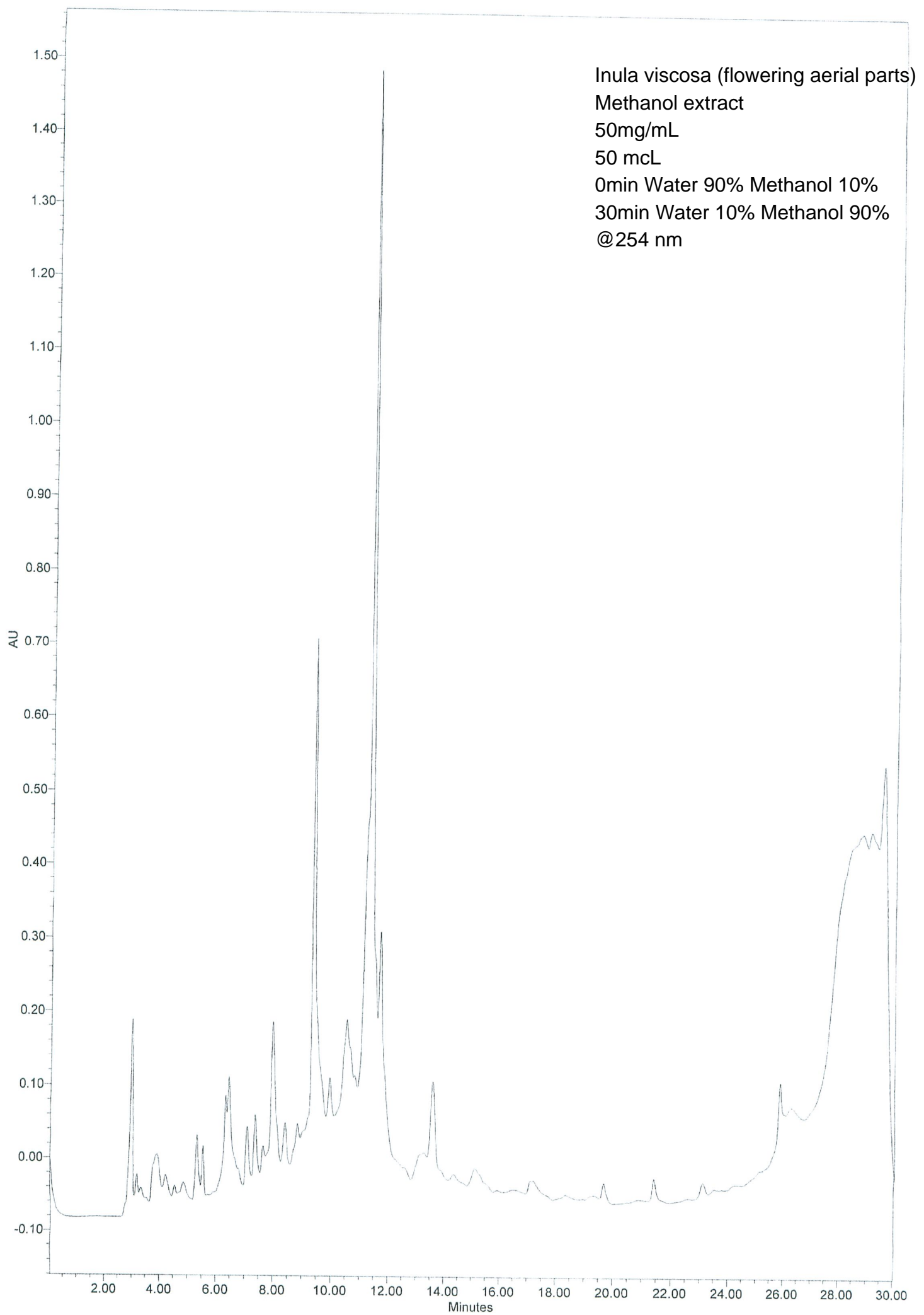

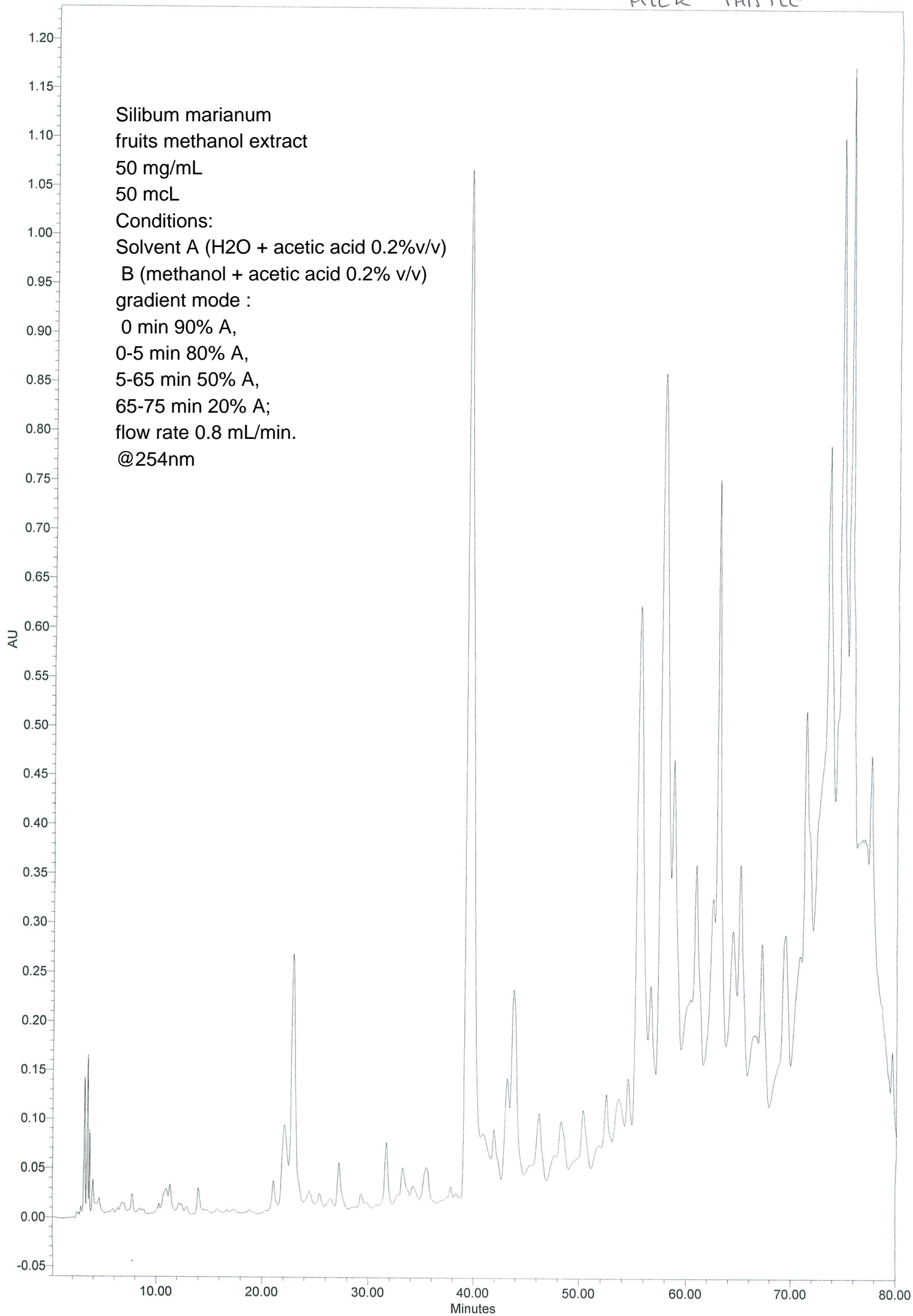

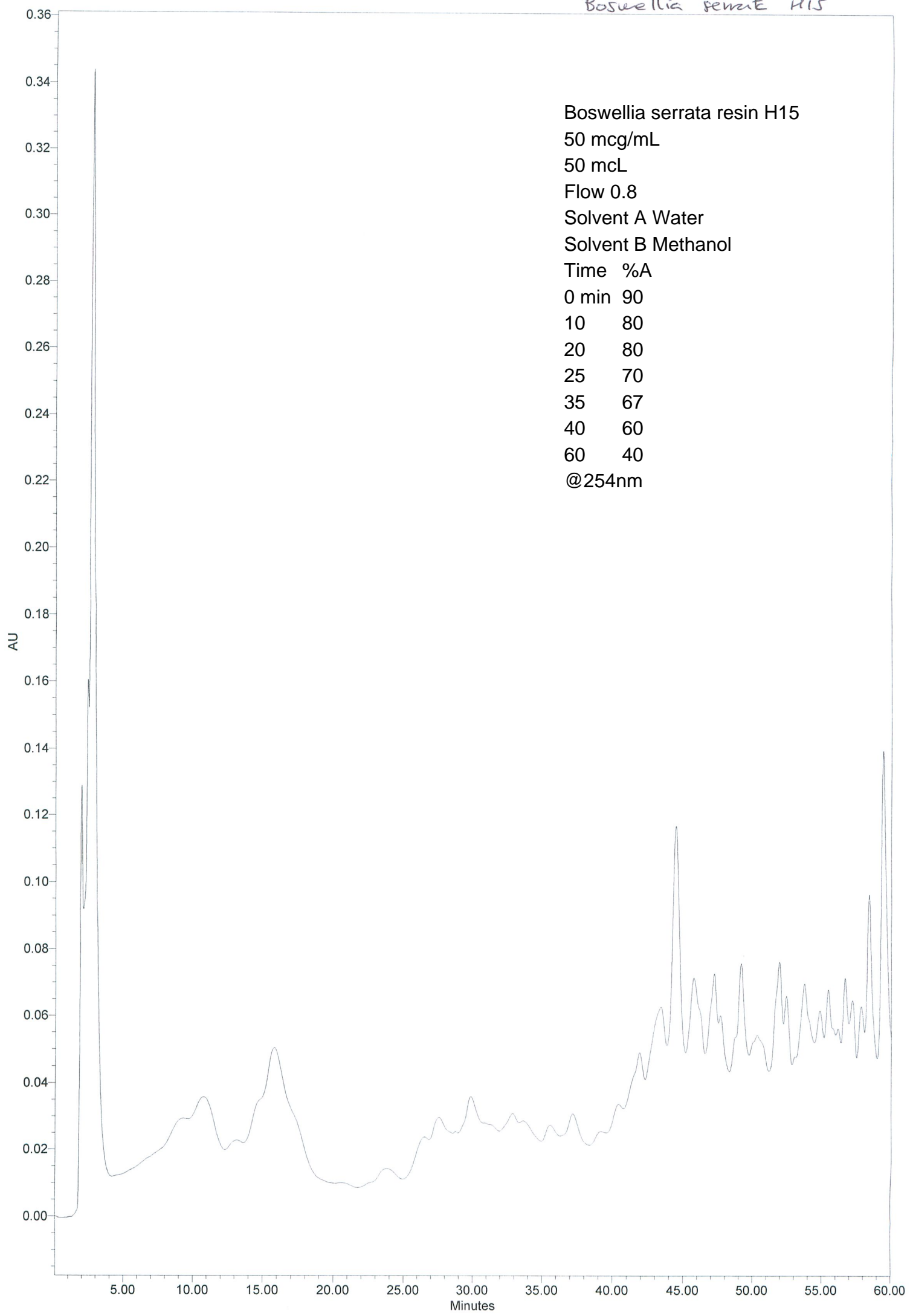

Supplement: Supplementary file 1 [file ptr0029-0108-sd1.pdf]
